# Indian Obstetrics

**Published:** 1878-08

**Authors:** 


### EXCERPTA.

### INDIAN OBSTETRICS.

E. B. Stevens, M.D.:

*Dear Doctor*—I have just received this, and it occurred to me it might be acceptable to the *Gazette* as a contribution report on Indian Midwifery.

W. H. Taylor.

Wichita Agency, Indian Territory, May 10, 1878.

Dr. Wm. H. Taylor, Miami Medical College, Cincinnati.

*Dear Friend*—I was called yesterday morning to an obstetrical case—occurring in the Pawnee camp. The child had just been born when I arrived, but a large tumor remained, and the husband suggested that there might be twins. On making examination I found the uterus quite distended with a soft, fluctuating body—not at all like a fœtus. There appearing want of uterine action, I gave quinine and ergot, and awaited the establishment of suitable contractions.

The woman was lying on her back, on a pallet elevated at an angle of about forty-five degrees, with her knees raised and her feet resting on the ground, in a half sitting, half reclining posture. When she began to feel the contractions she turned over on the couch, resting her knees and elbows at the angle named. I noticed her making some adjustments which I did not fully comprehend, and making an examination when a pain came on, I discovered there were two cords, and forcible traction was being made upon each of them.

Following these cords to their termination, I found them attached to two flattened stones, about the size of bricks, but thinner. Around these were tied pieces of cloth, and to each stone was attached the end of a cord. Upon the occurrence of each contraction these stones were so placed against the knees of the woman that she could, with a slight elevation of her body, draw upon the cords with as much or as little force as she desired.

The novelty and ingenuity of this method of making traction upon the cord forcibly impressed me with the ready wit of the Indian in adapting means to *ends* for each emergency.

The woman here becomes her own accouchuer, and hence the amount of traction is always proportionate to the quantity of strength she may have remaining to expend.

But in this case her tactics did not succeed, for I discovered that as she pressed upon the traction the blood would flow quite freely.

Inserting my hand into the uterus, I discovered that some adhesions existed between the placenta and walls of the uterus. Detaching these adhesions, I removed *two separate and distinct placentas*, each as large and as complete as an average placenta, and with a cord inserted in the centre of each. But one child was born, yet here appeared the accompaniments of two. The cord had been cut before I arrived, but the two cords appeared to unite near the umbilicous, some twenty-three or twenty-four inches from the placental insertions.

As in a similar case published by Cazeaux (fifth American edition) occurring in the practice of Dr. Ebert, the membranes formed a single cavity, and constituted the connecting link between the two placentas—these forming a kind of membranous bridge.

The extreme rarity of like recorded cases rendered this one exceedingly interesting to me. While I was studying upon this anomaly, the attendant squaws came in, and taking up the placentas, membranes, all soiled cloths, and even scraping the earth where blood had fallen upon it, wrapped all in a compact, oblong package, tightly corded, and carried it away for burial.

Other squaws came in with fresh earth and covered the floor where the couch was made, others brought in armfuls of freshly gathered weeds and placed them upon the earth; upon these the robes and blankets were spread, and the woman was comfortably disposed.—*Obstetric Gazette.*

